# Lactate orchestrates metabolic hemodynamic adaptations through a unique combination of venocontraction, artery relaxation, and positive inotropy

**DOI:** 10.1111/apha.70037

**Published:** 2025-04-01

**Authors:** Casper Homilius, Jacob M. Seefeldt, Jakob Hansen, Roni Nielsen, Frank V. de Paoli, Ebbe Boedtkjer

**Affiliations:** ^1^ Department of Biomedicine Aarhus University Aarhus Denmark; ^2^ Department of Clinical Medicine Aarhus University Aarhus Denmark; ^3^ Department of Cardiology Aarhus University Hospital Aarhus Denmark; ^4^ Department of Forensic Medicine Aarhus University Aarhus Denmark; ^5^ Department of Cardiothoracic and Vascular Surgery Aarhus University Hospital Aarhus Denmark

**Keywords:** blood flow, cardiac output, contractility, metabolites, preload, vascular resistance

## Abstract

**Aim:**

H^+^ facilitates metabolic blood flow regulation while negatively impacting cardiac contractility. Cardiovascular consequences of conjugate bases accumulating alongside H^+^ remain unclear. Here, we evaluate the cardiovascular effects of nine prominent carboxylates—particularly lactate, 3‐hydroxybutyrate, and butyrate—linked to metabolic and microbial activity.

**Methods:**

Comparing the actions of pH‐adjusted Na‐carboxylates to equiosmolar NaCl, we study arteries and veins isolated from healthy rats and humans with ischaemic heart disease, isolated perfused rat hearts, and rat cardiovascular function in vivo.

**Results:**

The tested carboxylates generally relax arteries and veins. L‐lactate relaxes human and rat arteries up to 70% (EC_50_ = 10.1 mM) and rat brachial and mesenteric veins up to 30% of pre‐contractions, yet stands out by augmenting contractions of rat femoral, saphenous, and lateral marginal veins and human internal thoracic and great saphenous veins up to 50%. D‐lactate shows only minor actions. In isolated perfused hearts, 10 mM L‐lactate increases coronary flow (17.1 ± 7.7%) and left ventricular developed pressure (10.1 ± 3.0%) without affecting heart rate. L‐lactate infusion in rats—reaching 3.7 ± 0.3 mM in the circulation—increases left ventricular end‐diastolic volume (11.3 ± 2.8%), stroke volume (22.6 ± 3.0%), cardiac output (23.4 ± 3.5%), and ejection fraction (10.6 ± 2.0%), and lowers systemic vascular resistance (34.1 ± 3.7%) without influencing blood pressure or heart rate. The ketone body 3‐hydroxybutyrate causes lactate accumulation and elevates left ventricular end‐diastolic volume in vivo.

**Conclusion:**

Carboxylate metabolites generally relax arteries and veins. L‐lactate relaxes arteries, lowering systemic vascular resistance, causes preferential venocontraction with increased ventricular diastolic filling, and elevates cardiac contractility and cardiac output. We propose that L‐lactate optimizes cardiovascular function during metabolic disturbances.

## 1 INTRODUCTION

Metabolites accumulate locally or systemically in response to altered metabolic state (e.g., fasting or metabolic disease), activity level, and in conditions of insufficient tissue perfusion. Whereas cardiovascular consequences of metabolic and respiratory acidosis have been studied for more than a century,^1^, ^2^ the functional effects of the conjugate bases that build up in parallel with H^+^ remain understudied. The accumulating conjugate bases are often carboxylate metabolites that reflect the cause of the acidosis, for example, lactate during circulatory failure, 3‐hydroxybutyrate during diabetic ketoacidosis, and HCO_3_^−^ during respiratory acidosis. Also, corresponding carboxylates of short‐chain fatty acids—for example, butyrate, propionate, and acetate—are released by the intestinal microbiota, providing a putative link between the microbial composition in the gut and cardiovascular health.^3^

Growing evidence supports that carboxylates derived from metabolism or the intestinal microbiota influence systemic hemodynamic functions.^4^, ^5^, ^6^, ^7^ Lactate exists as two (D‐ and L‐) enantiomers.^8^ L‐lactate is endogenously produced in humans during physiological challenges and pathophysiological conditions of elevated fermentative glycolysis. Production and accumulation of L‐lactate increase dynamically during periods of high metabolic demand (e.g., exercise) or low oxygen availability (e.g., ischaemia). Plasma L‐lactate concentrations typically range between 0.5 and 1 mM in healthy individuals under resting conditions.^9^, ^10^ During exercise and in the immediate recovery period, L‐lactate concentrations in the interstitial space of contracting muscles and in plasma rise swiftly to 5–10 mM, and they can peak as high as 25 mM in cases of extreme exertion.^9^, ^11^ D‐lactate, which is usually present at low micromolar concentration in human plasma, is produced by certain gut bacteria and in very small amounts through the glyoxalase pathway.^8^, ^12^

Whereas L‐lactate was classically considered a metabolic waste product, it is an attractive oxidative energy source,^13^ regulator of skeletal muscle excitability,^14^ and a signal for regulation of lipolysis and inflammation.^15^, ^16^ Earlier studies show that L‐lactate modulates arterial tone,^7^, ^17^, ^18^, ^19^, ^20^, ^21^, ^22^ but effects on the venous circulation have not previously been explored. Also, whereas previous investigations on isolated perfused hearts suggested that L‐lactate depresses left ventricular contractions,^22^, ^23^ recent observations challenge this perception and show that L‐lactate can enhance cardiac function in heart failure patients.^24^

Evaluating the influence of endogenous L‐lactate levels on the cardiovascular system to establish prognostic causality is difficult because L‐lactate is typically more prominently produced, and L‐lactate concentrations are therefore more markedly elevated in patients with critical disease. Thus, L‐lactate is a widely used biomarker indicating hypoperfusion and inadequate tissue oxygenation.^25^, ^26^, ^27^ Specifically, in patients with acute cardiac disease, elevated L‐lactate levels are associated with increased mortality, inferior responses to percutaneous coronary intervention,^28^ and larger infarct sizes.^29^ Even though accelerated endogenous L‐lactate production associates with poor clinical outcomes, the functional effects of lactate—and hence exogenous L‐lactate administration—may still improve cardiovascular performance. Supporting that critically ill patients are worse off without the cardiovascular influences of L‐lactate, studies show that exogenous L‐lactate can benefit patients with acute heart failure^24^ or undergoing intensive care after coronary artery bypass surgery.^30^

Based on similar influences of Na‐lactate and NaCl on temperature regulation and eating behavior, investigators recently argued that effects previously ascribed to lactate are largely consequences of osmolarity.^31^ With this in mind, well‐designed experimental studies—including carefully performed time and osmotic controls—are warranted to identify relevant cardiovascular adaptations to elevated lactate levels.

In the current study, we first screen a panel of metabolic and microbial carboxylates for actions on arterial and venous tone relative to equiosmolar NaCl. We then explore the effects of lactate on isolated arteries, veins, and hearts. We initially test the influences of lactate on rat tissue and then confirm the vascular responses in human blood vessels from patients undergoing coronary artery bypass grafting (CABG) or J‐incision off pump coronary artery bypass (JOPCAB). We further test the in vivo integrated hemodynamic effects of L‐lactate administration in rats and compare them to those of butyrate and DL‐3‐hydroxybutyrate. We demonstrate that L‐lactate elevates cardiac output through (a) preferential venocontraction and increased left ventricular preload, (b) arterial relaxation and reduced systemic vascular resistance, and (c) enhanced cardiac contractility.

## 2 RESULTS

**FIGURE 1:**
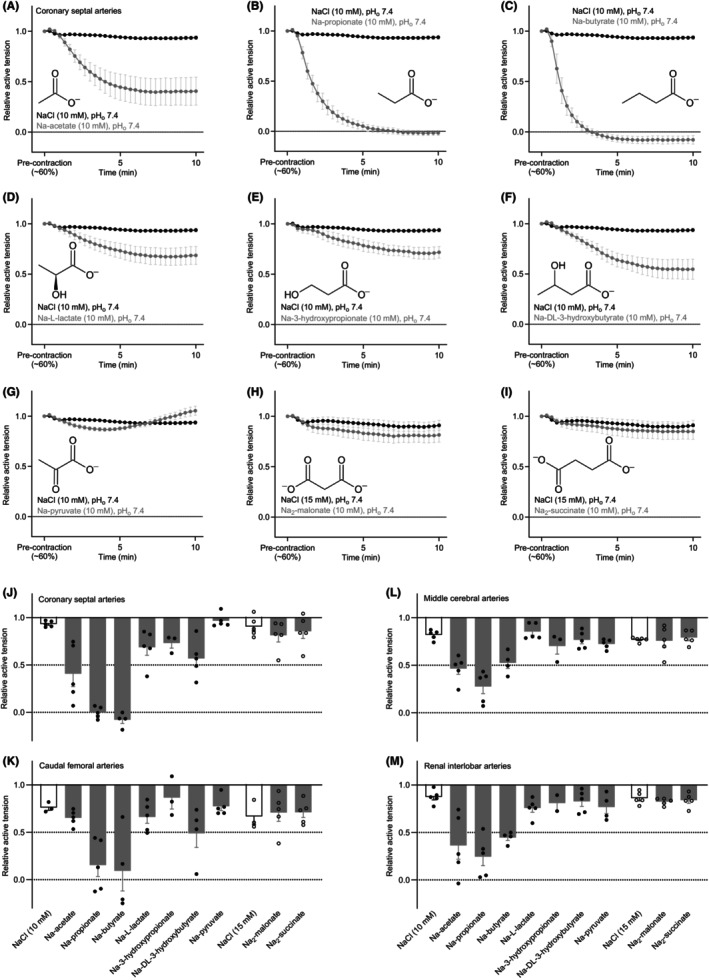
Metabolic and microbial carboxylates relax rat arteries. The arterial responses are shown relative to a stable U46619‐induced pre‐contraction corresponding to around 60% of the initial maximal contraction. (A–I) Average vasomotor responses of rat coronary septal arteries mounted for evaluation of isometric tension development and stimulated with 10 mM of one of nine biologically prominent sodium carboxylates. For comparison, each panel repeats the response to equiosmolar NaCl (*n* = 3–5). (J–N) Relative changes in coronary septal artery (J), caudal femoral artery (K), middle cerebral artery (L), and renal interlobar artery (M) tone in response to application of 10 mM sodium carboxylate or equiosmolar NaCl. In all cases, the pH of the buffer in the myograph bath was maintained by titrating the carboxylate‐containing solutions to pH 7.40 before application. Arterial responses were averaged over the last 5 min of sodium carboxylate or additional NaCl exposure.

**FIGURE 2:**
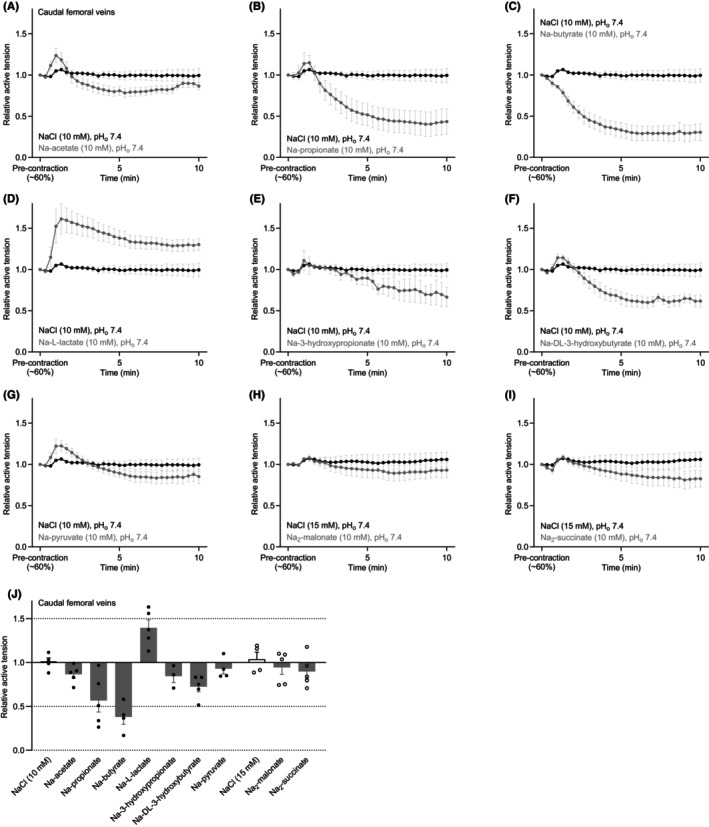
Metabolic and microbial carboxylates generally relax rat caudal femoral veins; however, L‐lactate causes venocontraction. The venous responses are shown relative to a stable U46619‐induced pre‐contraction corresponding to around 60% of the initial maximal contraction. (A–I) Average responses of caudal femoral veins mounted for evaluation of isometric tension development and stimulated with 10 mM of one of nine biologically prominent sodium carboxylates. For comparison, each panel repeats the response to equiosmolar NaCl (*n* = 3–5). (J) Relative changes in caudal femoral venous tone in response to the application of 10 mM sodium carboxylate or equiosmolar NaCl. In all cases, the pH of the buffer in the myograph bath was maintained by titrating the carboxylate‐containing solutions to pH 7.40 before application. Venous responses were averaged over the last 9 min of sodium carboxylate or additional NaCl exposure.

We first screened arterial and venous responses to nine biologically prominent and structurally related carboxylates: the hydroxycarboxylates L‐lactate (product of fermentative glycolysis), DL‐3‐hydroxybutyrate (ketone body), and 3‐hydroxypropionate; the ketocarboxylate pyruvate (product of glycolysis); the conjugate bases acetate, propionate, and butyrate of microbiota‐derived short‐chain fatty acids; and the dicarboxylates malonate and succinate from the tricarboxylic acid cycle. We evaluated influences of these carboxylates on rat coronary septal (Figure 1A–J), caudal femoral (Figure S1 and Figure 1K), middle cerebral (Figure S2 and Figure 1L), and renal interlobar (Figure S3 and Figure 1M) arteries as well as caudal femoral veins (Figure 2) mounted in wire myographs and pre‐contracted with the thromboxane analog U46619. Table S1 reports the diameters and the maximal tension development for each of the evaluated blood vessel types.

### 2.1 Metabolic and microbial carboxylates cause arterial vasorelaxation

Relative to equiosmolar NaCl, we observed the strongest and most widespread arterial vasomotor responses to Na‐acetate (Figure 1A), Na‐propionate (Figure 1B), and Na‐butyrate (Figure 1C) that relaxed coronary septal arteries (Figure 1J), caudal femoral arteries (Figure 1K), middle cerebral arteries (Figure 1L), and renal interlobar arteries (Figure 1M). The responses to 10 mM of the three microbiota‐derived carboxylates varied in magnitude among the tested arteries with full relaxation of active tone achieved when butyrate was applied to coronary septal and caudal femoral arteries (Figure 1J–M). Acetate showed a smaller response than propionate and butyrate in coronary (Figure 1A–C,J) and skeletal muscle (Figure 1K) arteries, whereas responses to the three short‐chain fatty acids were more similar in cerebral (Figure 1L) and renal (Figure 1M) arteries.

The magnitude of the vasorelaxant response to 10 mM DL‐3‐hydroxybutyrate followed a profile between arterial beds consistent with previous reports.^5^ The greatest vasorelaxation was observed in coronary septal and caudal femoral arteries (Figure 1F,J,K), whereas the response was smaller in middle cerebral and renal interlobar arteries (Figure 1L,M). At 10 mM, 3‐hydroxypropionate caused a somewhat blunted vasorelaxant response compared to DL‐3‐hydroxybutyrate (Figure 1E,J–M), whereas arterial vasorelaxation in response to L‐lactate was most prominent in coronary septal arteries (Figure 1D,J–M).

Across the tested arterial beds, we observed very little vasomotor response to 10 mM Na‐pyruvate (Figure 1G,J–M) and only minor relaxations to the sodium salts of the dicarboxylates malonate and succinate (Figure 1H–M) when compared to equiosmolar NaCl.

The supporting information file includes average traces of the vasoactive responses from caudal femoral (Figure S1), middle cerebral (Figure S2), and renal interlobar (Figure S3) arteries.

### 2.2 Metabolic and microbial carboxylates generally cause venorelaxation, but L‐lactate can cause venocontraction

The responses of caudal femoral veins to 10 mM of the nine selected carboxylates were in many regards similar to the arterial responses (compare Figures 1 and 2). As such, acetate, propionate, and butyrate as well as DL‐3‐hydroxybutyrate (Figure 2A–C,F,J) all caused substantial relaxation of caudal femoral veins pre‐contracted with U46619. 3‐hydroxypropionate also induced considerable venorelaxation (Figure 2E,J), whereas pyruvate, malonate, and succinate produced relatively smaller venorelaxation (Figure 2G–J). In sharp contrast, however, L‐lactate enhanced the contraction of caudal femoral veins (Figure 2D,J).

The unique profile of L‐lactate—showing combined arterial relaxation and venous contraction—spurred us to analyze in greater detail how L‐lactate influences the cardiovascular system.

### 2.3 L‐lactate causes generalized arterial vasorelaxation at physiologically relevant concentrations

**FIGURE 3:**
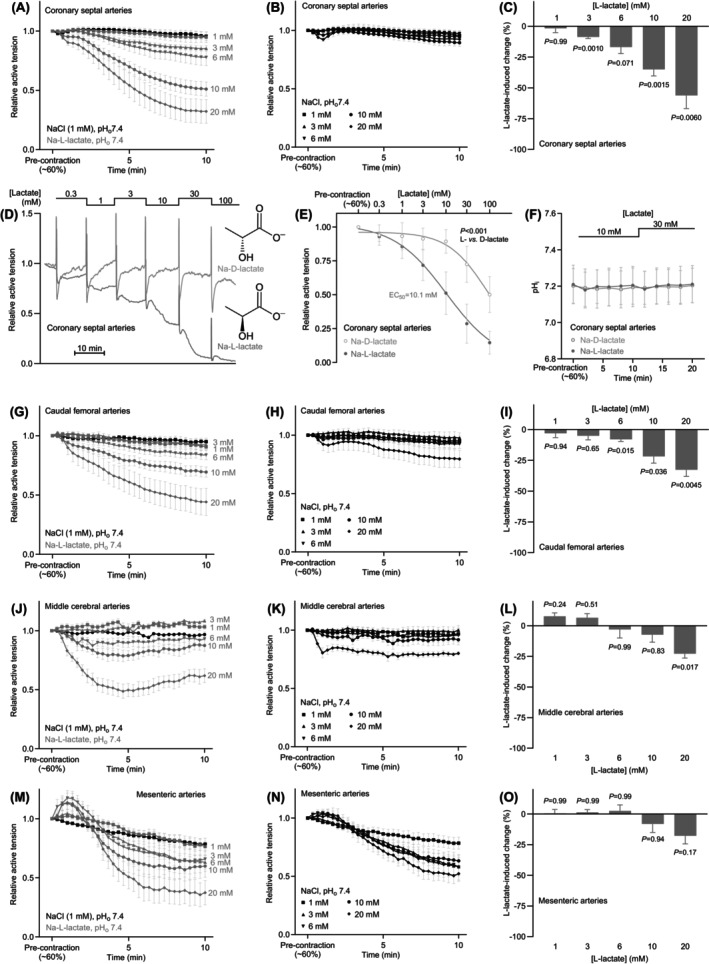
L‐lactate relaxes a broad range of rat arteries and shows an EC_50_ of 10.1 mM in coronary septal arteries. The arterial responses are shown relative to a stable U46619‐induced pre‐contraction corresponding to around 60% of the initial maximal contraction. (A, G, J, M) Average concentration‐dependent Na‐L‐lactate‐induced changes in rat coronary septal (A, *n* = 8), caudal femoral (G, *n* = 7), middle cerebral (J, *n* = 5), and mesenteric (M, *n* = 7) artery tone. (B, H, K, N) Average concentration‐dependent NaCl‐induced changes in rat coronary septal (B, *n* = 8), caudal femoral (H, *n* = 7), middle cerebral (K, *n* = 5), and mesenteric (N, *n* = 7) artery tone. (C, I, L, O) L‐lactate‐induced changes in rat coronary septal (C, *n* = 8), caudal femoral (I, *n* = 7), middle cerebral (L, *n* = 5), and mesenteric (O, *n* = 7) artery tone when adjusted for the response to equimolar NaCl. Data were compared by repeated‐measures two‐way ANOVA followed by Šídák's posttests. (D, E) Representative traces (D) and average concentration‐response curves (E, *n* = 5) that compare the vasomotor responses of coronary septal arteries to L‐lactate and D‐lactate. Sigmoidal curves were fitted to the data and compared by an extra sum‐of‐squares *F*‐test. (F) Intracellular pH (pH_i_) measured from coronary septal arteries during successive application of 10 and 30 mM Na‐L‐lactate or Na‐D‐lactate (*n* = 5). In all cases, the pH of the buffer in the myograph bath was maintained by titrating the carboxylate‐containing solutions to pH 7.40 before application. Arterial responses were averaged over the last 5 min of sodium carboxylate or additional NaCl exposure. The *p*‐values refer to comparisons versus 0, unless otherwise stated.

We first expanded our evaluation of L‐lactate to other systemic vascular beds and tested responses at multiple concentrations to establish the potential physiological and pathophysiological relevance (Figure 3).

We initially added Na‐L‐lactate to the standard physiological saline solution (PSS) and compared the vasomotor response to that elicited by a similar osmotic challenge from equimolar NaCl (Figure 3A,B). Based on this approach, L‐lactate caused concentration‐dependent vasorelaxation of coronary septal arteries that became evident at concentrations around 3 mM and reached a magnitude of around 60% of the U46619‐induced pre‐contraction when applied at a concentration of 20 mM (Figure 3A–C).

Next, to produce a more complete concentration–response relationship, we substituted Na‐L‐lactate for NaCl while keeping the osmolarity fixed at physiological level (Figure 3D). This approach revealed an EC_50_ of 10.1 mM for L‐lactate with regard to relaxation of coronary septal arteries (Figure 3E). We also used the same approach to test the response to D‐lactate, which showed a much less potent response (Figure 3D,E).

When tested at 10 and 30 mM—administered as solutions adjusted to pH 7.40—neither L‐lactate nor D‐lactate caused a sustained change in intracellular pH (pH_i_) of the vascular smooth muscle cells in the wall of coronary septal arteries (Figure 3F). We observed a small transient acidification to L‐lactate consistent with coupled H^+^ and lactate import via monocarboxylate transporters (Figure 3F); however, the immediate effect of L‐lactate on pH_i_ was only of −0.031 ± 0.008 magnitude (*p* = 0.012) and was compensated within 2 min (Figure 3F). Thus, vascular smooth muscle cell pH_i_ was unaffected in the interval from 5 to 10 min after lactate addition (Figure 3F) when arterial vasomotor responses were evaluated.

As shown in Figure 3G–O, higher concentrations of L‐lactate also relaxed caudal femoral (up to 33 ± 5%), middle cerebral (up to 23 ± 4%), and mesenteric (up to 18 ± 7%) arteries, but the responses were of smaller magnitude than the relaxation of coronary septal arteries (up to 56 ± 11%) in the concentration range to 20 mM.

### 2.4 L‐lactate causes preferential venocontraction at physiological concentrations

**FIGURE 4:**
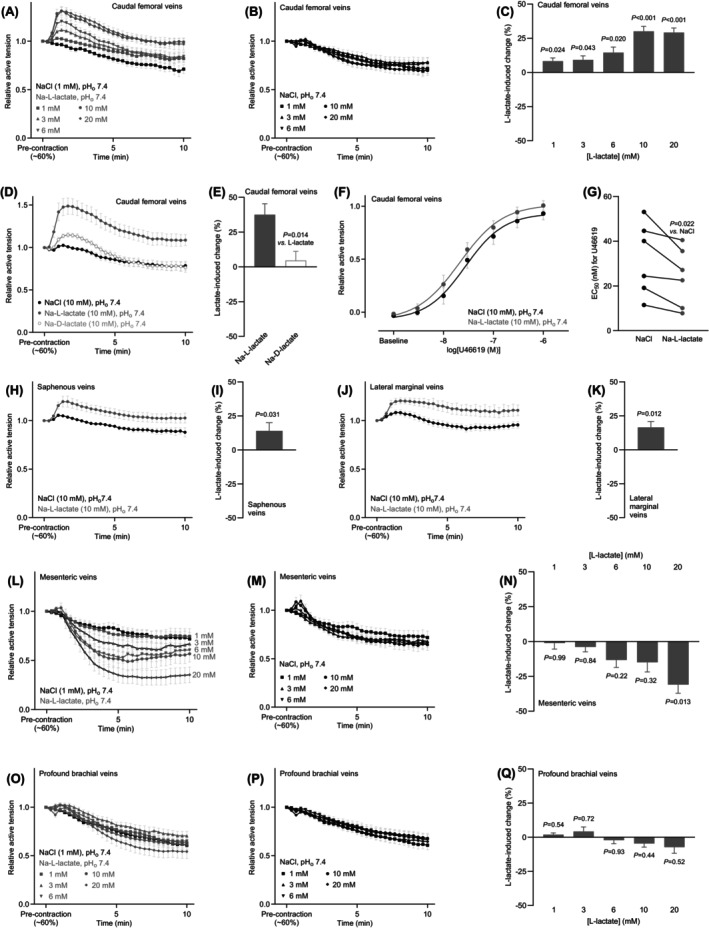
L‐lactate contracts hindlimb rat veins. The venous responses are shown relative to a stable U46619‐induced pre‐contraction corresponding to around 60% of the initial maximal contraction. (A, B, H, J, L, M, O, P) Average changes in caudal femoral (A, B, *n* = 12), saphenous (H, *n* = 6), lateral marginal (J, *n* = 6), mesenteric (L, M, *n* = 7), and profound brachial (O, P, *n* = 11) venous tone in response to Na‐L‐lactate or equimolar NaCl. (C, I, K, N, Q) L‐lactate‐induced changes in caudal femoral (C, *n* = 12), saphenous (I, *n* = 6), lateral marginal (K, *n* = 6), mesenteric (N, *n* = 7), and profound brachial (Q, *n* = 11) venous tone when adjusted for the response to equimolar NaCl. Data in panels C, N, and Q were compared by repeated‐measures two‐way ANOVA followed by Šídák's posttest, data in panel I were compared by the nonparametric Wilcoxon matched‐pairs signed rank test, and data in panel K were compared by paired two‐tailed Student's *t*‐test. (D) Average changes in caudal femoral venous tone (*n* = 6) in response to 10 mM Na‐L‐lactate, Na‐D‐lactate, or NaCl. (E) L‐lactate‐ and D‐lactate‐induced changes in caudal femoral venous tone (*n* = 6) when adjusted for the response to equimolar NaCl. The lactate enantiomers were tested at 10 mM. Data were compared by paired two‐tailed Student's *t*‐test. (F, G) Concentration–response relationships (F) and corresponding EC_50_ values (G) for caudal femoral venous contractions elicited with U46619 in PSS added 10 mM Na‐L‐lactate or equimolar extra NaCl (*n* = 6). The veins were preincubated with L‐lactate or additional NaCl for 3 min before addition of the first concentration of U46619. Sigmoidal curves were fitted to the data and the derived EC_50_ values compared by paired two‐tailed Student's *t*‐test. In all cases, the pH of the buffer in the myograph bath was maintained by titrating the carboxylate‐containing solutions to pH 7.40 before application. Venous responses were averaged over the last 9 min of sodium carboxylate or additional NaCl exposure. The *p*‐values refer to comparisons versus 0, unless otherwise stated.

L‐lactate concentration‐dependently contracted caudal femoral veins and even in the low physiological range around 1 mM caused small augmentations of the U46619‐induced pre‐contractions (Figure 4A–C). When applied at 10 mM, L‐lactate induced a very substantial contraction, whereas D‐lactate caused only a modest transient response (Figure 4D,E). Although 10 mM L‐lactate alone did not elevate basal tone of caudal femoral veins, we observed an accentuation of the contractile response to U46619 when the veins were preincubated with 10 mM L‐lactate (Figure 4F,G).

Other hind limb veins pre‐contracted with U46619—we tested saphenous and lateral marginal veins—also elevated their tension development when stimulated with 10 mM L‐lactate (Figure 4H–K). In contrast, when compared to equimolar NaCl, L‐lactate caused modest relaxation of mesenteric veins, reaching statistical significance only at 20 mM (Figure 4L–N), and showed no overall effect on the tone of profound brachial veins (Figure 4O–Q).

### 2.5 Human arteries relax and veins contract in response to L‐lactate

To facilitate translation of our findings from healthy rats to humans with ischaemic heart disease, we next obtained segments of human arteries and veins from patients undergoing CABG or JOPCAB. The patient cohort is described in Table 1. We compared the effects of L‐lactate to those of NaCl and also tested DL‐3‐hydroxybutyrate, which was previously found to have beneficial hemodynamic effects in healthy humans and patients suffering from pulmonary hypertension, heart failure with reduced ejection fraction, or cardiogenic shock.^32^, ^33^, ^34^, ^35^ All human blood vessels were pre‐contracted with U46619 before the addition of the carboxylate metabolites.

**TABLE 1:** Clinical characteristics of the patients included in this study. All patients were male and underwent traditional coronary artery bypass grafting (89%; CABG) or J‐incision off‐pump coronary artery bypass (11%; JOPCAB) at Aarhus University Hospital, Denmark. We report mean ± SD, unless otherwise specified. PRN, pro re nata/as needed.

Number of patients	9
Age (years)	66.6 ± 8.0
Height (cm)	176 ± 4
Body weight (kg)	86 ± 12
Body mass index (kg/m^2^)	27.8 ± 3.6
Left ventricular ejection fraction	54 ± 10%
Estimated glomerular filtration rate (mL/min; median [interval])	82 [61, >90]
*Diagnoses (cardiovascular and metabolic)*	
Ischaemic heart disease	100%
Essential hypertension	56%
Hypercholesterolemia	56%
Type‐2 diabetes mellitus	22%
Heart failure	22%
Orthostatic hypotension	11%
Acute myocardial infarction (NSTEMI)	11%
Paroxysmal atrial fibrillation	11%
Claudication	11%
Elevated lipoprotein (a)	11%
*Medication*	
Statin (atorva‐, simva‐, and rosuvastatin)	100%
Acetylsalicylic acid	89%
L‐type Ca^2+^‐channel blocker (amlodipine)	56%
Angiotensin II type 1 receptor blocker (losartan)	56%
β_1_‐adrenoceptor blocker (carvedilol, metoprolol)	44%
Nitroglycerin (PRN)	44%
Sodium glucose transporter‐2 (SGLT2) inhibitor (dapagliflozin)	33%
Paracetamol	33%
Metformin	22%
Angiotensin converting enzyme inhibitor (enalapril)	22%
Thiazide diuretic	22%
Loop diuretic (furosemide)	22%
P_2_Y_12_‐receptor antagonist (ticagrelor)	22%
Cholesterol absorption inhibitor (ezetimibe)	22%
B12 supplement	11%
Antihistamine (cetirizine)	11%
Xanthine oxidase inhibitor (allopurinol)	11%
Phosphodiesterase 5 inhibitor (sildenafil; PRN)	11%
Selective serotonin reuptake inhibitor (paroxetine)	11%
Factor Xa inhibitor (rivaroxaban)	11%
Amiodarone	11%

**FIGURE 5:**
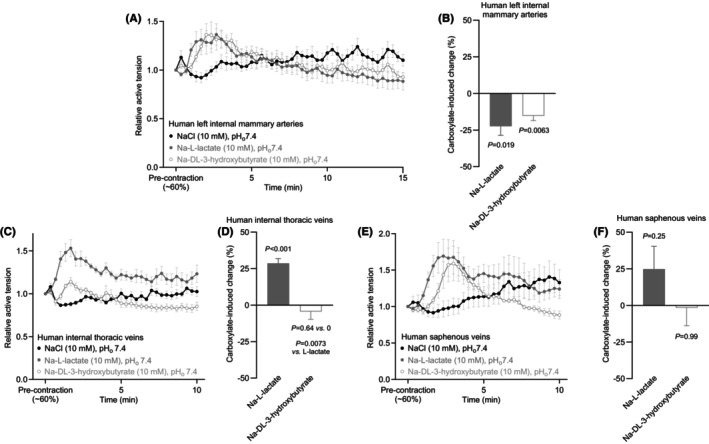
L‐lactate and DL‐3‐hydroxybutyrate both relax human left internal mammary arteries, whereas only L‐lactate contracts human internal thoracic veins from patients with ischaemic heart disease. The vascular responses are shown relative to a stable U46619‐induced pre‐contraction corresponding to at least 20% of the initial maximal contraction. (A, C, E) Average changes in human left internal mammary artery (A, *n* = 7), human internal thoracic venous (C, *n* = 7), and human saphenous venous (E, *n* = 8) tone in response to 10 mM Na‐L‐lactate, Na‐DL‐3‐hydroxybutyrate, and NaCl. (B, D, F) L‐lactate‐ and DL‐3‐hydroxybutyrate‐induced changes in human left internal mammary artery (B, *n* = 7), human internal thoracic venous (D, *n* = 7), and human saphenous venous (F, *n* = 8) tone when adjusted for the response to equimolar NaCl. The carboxylates were tested at 10 mM. Data were compared by repeated measures one‐way ANOVA followed by Dunnett or Šídák's post‐tests. In all cases, the pH of the buffer in the myograph bath was maintained by titrating the carboxylate‐containing solutions to pH 7.40 before application. Arterial responses were averaged over the last 5 min, and venous responses over the last 9 min of sodium carboxylate or additional NaCl exposure. The *p*‐values refer to comparisons versus 0, unless otherwise stated.

After a transient initial contraction, human left internal mammary arteries relaxed to an approximately similar extent in response to L‐lactate and DL‐3‐hydroxybutyrate (Figure 5A,B).

As illustrated in Figure 5C,D, human internal thoracic veins markedly contracted to L‐lactate, yet not to DL‐3‐hydroxybutyrate, confirming the unique vascular profile of L‐lactate also in humans. Although human saphenous veins responded to L‐lactate, DL‐3‐hydroxybutyrate, and NaCl (Figure 5E) with a contractile profile that resembled that of human internal thoracic veins (Figure 5C), the responses of these large‐size saphenous veins showed greater variability, and the differences did not reach statistical significance (Figure 5F).

### 2.6 L‐lactate increases contractility and coronary flow in isolated perfused hearts

**FIGURE 6:**
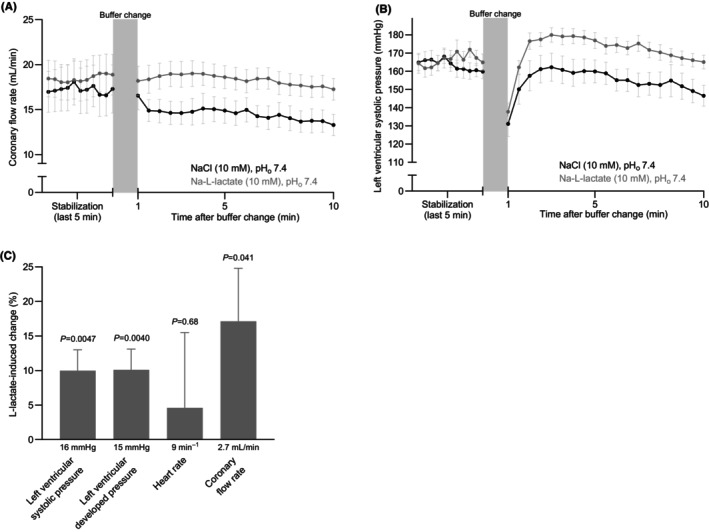
L‐lactate increases cardiac contractility and coronary flow rate in isolated perfused hearts. (A, B) Average traces of coronary flow rate (A) and left ventricular systolic pressure (B) during perfusion with Krebs–Henseleit buffer (stabilization) and after the addition of 10 mM Na‐L‐lactate or 10 mM extra NaCl (*n* = 8–10). Note that a washing artifact is observed related to the buffer change and that it is followed by a modest and slow gradual rundown over time. (C) L‐lactate‐induced changes in cardiac variables 10 min after the buffer change when adjusted for the response to equimolar NaCl (*n* = 8–10). In addition to the relative changes illustrated by the bars, the values at the base of the bars indicate the absolute changes. Data were compared by unpaired two‐tailed Student's *t*‐tests. The standard and L‐lactate‐containing Krebs–Henseleit buffers were both titrated to pH 7.40. The *p*‐values refer to comparisons versus 0.

We evaluated the effects of L‐lactate on coronary flow rate and contractile function in isolated perfused rat hearts mounted in Langendorff systems with fixed pre‐ and afterload (Figure 6). We compared the effects of L‐lactate to equimolar NaCl added to the pH‐adjusted perfusion solutions. We observed no differences in baseline parameters between the compared groups of isolated perfused hearts (Table S2).

Relative to equiosmolar NaCl, perfusion with 10 mM L‐lactate increased coronary flow rate by 17.1 ± 7.7% (Figure 6A,C), left ventricular systolic pressure by 10.0 ± 3.0% (Figure 6B,C), and left ventricular developed pressure by 10.1 ± 3.0% (Figure 6C) whereas heart rate was unaffected (Figure 6C). These findings support that L‐lactate increases cardiac contractility.

### 2.7 L‐lactate increases cardiac filling and contractility and lowers systemic vascular resistance

We next explored the in vivo cardiac and hemodynamic effects of continuous, intravenous infusion of Na‐L‐lactate and compared the response to equimolar NaCl infused at a similar rate. We observed no difference in baseline parameters between rats in the treatment and control groups used for echocardiography or blood pressure measurements (Table S3).

**FIGURE 7:**
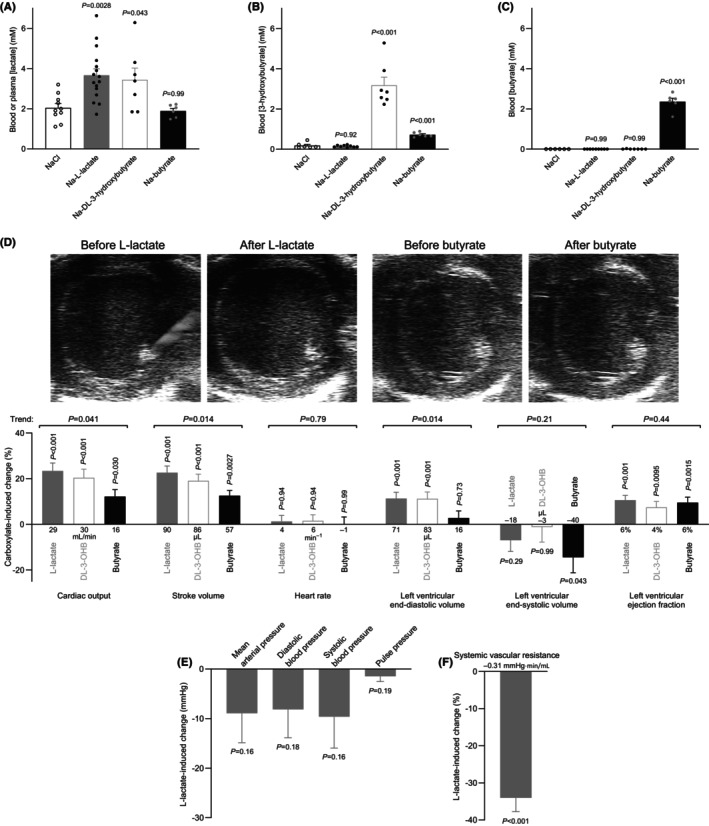
L‐lactate infusion in vivo elevates cardiac output, stroke volume, left ventricular diastolic filling, and ejection fraction and lowers systemic vascular resistance. Administration of DL‐3‐hydroxybutyrate, but not butyrate, causes endogenous lactate accumulation in vivo with associated hemodynamic adaptations. (A–C) Plasma or blood concentrations of lactate (A), 3‐hydroxybutyrate (B), and butyrate (C) after infusion of 1 M Na‐L‐lactate, Na‐DL‐3‐hydroxybutyrate, Na‐butyrate, or NaCl at a rate of 10 mL/h per kg body weight for 10 minutes (*n* = 6–16). (D) L‐lactate‐, DL‐3‐hydroxybutyrate (DL‐3‐OHB)‐, and butyrate‐induced changes in echocardiographic variables (*n* = 6–15) when adjusted for the response to equimolar NaCl. The echocardiographic images were captured at the end of diastole. In addition to the relative changes illustrated by the bars, the values at the base of the bars indicate the absolute changes. (E, F) L‐lactate‐induced changes in blood pressure (E, *n* = 6–7) and systemic vascular resistance (F, *n* = 6–7) when adjusted for the response to equimolar NaCl. In addition to the relative change in systemic vascular resistance illustrated by the bar, the value at the base of the bar indicates the absolute change. Data in panels A–D were compared by one‐way ANOVA followed by Dunnett's posttest or posttest for trend. Data in panel E and F were compared by unpaired two‐tailed Student's *t*‐tests. The injection solutions were adjusted to pH 7.0 before intravenous administration. The *p*‐values refer to comparisons versus NaCl (panel A–C) or versus 0 (panel D–F) or report linear trend (panel D).

Intravenous infusion of Na‐L‐lactate increased the circulating lactate concentrations to 3.7 ± 0.3 mM compared with 2.0 ± 0.2 mM for rats infused with NaCl (Figure 7A).

The infusion solutions were adjusted to pH 7.0 before administration. Cellular uptake of lactate occurs usually via monocarboxylate transporters in symport with H^+^. As a likely consequence, we observed that Na‐L‐lactate infusion—even when administered at this slightly reduced pH compared to blood—modestly alkalinized the arterial blood by 0.047 ± 0.014 (*n* = 4–7, *p* = 0.0064) compared to NaCl infusion.

Based on our echocardiographic evaluation, L‐lactate elevated cardiac output by 23.4 ± 3.5% relative to the isoosmotic NaCl control, and this effect was related to an increase in stroke volume of 22.6 ± 3.0%, whereas heart rate remained unchanged (Figure 7D). The elevation of stroke volume was mainly related to an 11.3 ± 2.8% increase in end‐diastolic volume, whereas end‐systolic volume showed no significant change (Figure 7D). Accordingly, left ventricular ejection fraction increased by 10.6 ± 2.0% (Figure 7D).

From the invasive blood pressure measurements, we found that infusion of L‐lactate did not significantly change mean arterial blood pressure, pulse pressure, systolic, or diastolic blood pressure, although a tendency for reduction across all variables was apparent (Figure 7E).

Based on the animals where cardiac output and mean arterial pressure were measured simultaneously, we calculated an L‐lactate‐induced reduction in systemic vascular resistance of 34.1 ± 3.7% (Figure 7F).

### 2.8 L‐lactate concentrations and cardiac filling increase in response to the administration of DL‐3‐hydroxybutyrate but not butyrate

We finally evaluated whether the distinct profiles of arterial and venous vasomotor action elicited by L‐lactate, DL‐3‐hydroxybutyrate, and butyrate (Figures 1, 2, 3, 4, 5 and summarized in Figure S4A–C) translate to different in vivo cardiovascular consequences. We used the same infusion protocol (1 M, pH 7.0, at a rate of 10 mL/h per kg body weight for 10 minutes) for NaCl and the three carboxylates—thereby reaching blood and plasma concentrations of 3.7 ± 0.3 mM lactate (Figure 7A), 3.2 ± 0.4 mM 3‐hydroxybutyrate (Figure 7B), and 2.4 ± 0.2 mM butyrate (Figure 7C)—and compared their effects on echocardiographic variables (Figure 7D). Notably, infusion of DL‐3‐hydroxybutyrate caused accumulation of endogenous lactate, which increased from 2.0 ± 0.2 mM in NaCl‐infused rats to 3.4 ± 0.6 mM (Figure 7A), similar to the levels reached when L‐lactate was applied exogenously. Infusion of butyrate caused a modest increase in 3‐hydroxybutyrate concentration to 0.72 ± 0.05 mM compared with 0.18 ± 0.06 mM in NaCl‐infused rats (Figure 7B). Otherwise, the separate infusions of L‐lactate, DL‐3‐hydroxybutyrate, and butyrate did not mutually affect concentrations of the other carboxylates (Figure 7A–C).

The increase in left ventricular end‐diastolic volume (11.3 ± 2.8%, Figure 7D) upon in vivo L‐lactate infusion is consistent with its venocontractile influence (Figures 2D and 4). Substantiating the importance of venocontraction for the greater ventricular diastolic filling during L‐lactate administration, infusion of the venorelaxant butyrate did not similarly affect left ventricular end‐diastolic volume (2.8 ± 3.1%, Figure 7D). Although the elevated left ventricular end‐diastolic volume (11.2 ± 2.9%, Figure 7D) in response to DL‐3‐hydroxybutyrate infusion is surprising considering its direct venorelaxant effect ex vivo (Figure 2F), this observation is consistent with the accumulation of the venocontractile L‐lactate during ketosis induced by DL‐3‐hydroxybutyrate administration (Figure 7A).

Our echocardiographic evaluation shows almost identical effects of L‐lactate and DL‐3‐hydroxybutyrate infusions with regard to the increase in cardiac output, stroke volume, and ejection fraction (Figure 7D). In line with the lower preload in rats treated with butyrate compared to L‐lactate or DL‐3‐hydroxybutyrate, butyrate showed a pattern of less augmented cardiac output and stroke volume relative to the other carboxylates (Figure 7D). The increase in ejection fraction during butyrate infusion was of similar magnitude as that during L‐lactate and DL‐3‐hydroxybutyrate administration; but in the case of butyrate, it was driven by a decrease in end‐systolic volume rather than an increase in end‐diastolic volume (Figure 7D). None of the carboxylate infusions showed any effect on heart rate (Figure 7D).

Taken together, the trend from venocontraction to venorelaxation in response to L‐lactate, DL‐3‐hydroxybutyrate, and butyrate (Figures 2 and 4, and Figure S4) is reflected in similar trends in the magnitude of change in end‐diastolic volume, stroke volume, and cardiac output (Figure 7D). Hence, our observations support that the venocontractile effect of L‐lactate shifts venous blood volume toward the heart and that the consequent elevation of preload—in combination with a positive inotropic effect—augments stroke volume and cardiac output.

## 3 DISCUSSION

Here, we demonstrate the cardiovascular impact of a variety of carboxylates linked to metabolic and microbial activity. Although carboxylate metabolites generally induce combined arterial and venous relaxation (Figures 1 and 2), L‐lactate stands out as it relaxes arteries (Figures 1, 3, and 5) but preferentially contracts veins (Figures 2, 4, and 5) from both healthy rats and humans with ischaemic heart disease. In rats, L‐lactate causes pronounced contraction of hind limb veins (Figures 2 and 4); accordingly, in vivo infusion of L‐lactate redistributes the blood volume to elevate cardiac diastolic filling (Figure 7D). Consistent with the L‐lactate‐induced increase in left ventricular end‐diastolic volume (Figure 7D) and a direct elevation of cardiac contractility—as evidenced by higher systolic developed pressure of isolated perfused hearts (Figure 6B,C) and elevated left ventricular ejection fraction in vivo (Figure 7D)—L‐lactate increases cardiac output and stroke volume by around 25% without significantly influencing heart rate (Figure 7D). The simultaneous and roughly proportional decrease in systemic vascular resistance (Figure 7F) is consistent with the direct relaxant influence of L‐lactate on arteries (Figures 1, 3, and 5) and explains that mean arterial blood pressure remains unperturbed (Figure 7E).

L‐lactate‐induced arterial relaxations and venous contractions ex vivo are concentration‐dependent and occur within the physiologically and pathophysiologically relevant concentration ranges (Figures 3C,E,I and 4C). L‐lactate relaxes coronary septal arteries with an EC_50_ of 10.1 mM (Figure 3E) and contracts caudal femoral veins even at 1 mM (Figure 4C). Whereas L‐lactate significantly relaxes coronary septal arteries at 3 mM (Figure 3C), relaxation of caudal femoral and middle cerebral arteries requires 6 and 20 mM (Figure 3I,L), respectively, and we observe no significant vasomotor effect of even 20 mM L‐lactate on mesenteric arteries (Figure 3O). Notably, among the tested arteries, the mesenteric arteries show the greatest vasomotor actions to osmotic challenges (Figure 3N). The uneven pattern of vasorelaxant responsiveness to L‐lactate across the tested arterial beds is expected to divert blood flow to the heart and active skeletal muscles during intense exercise and to augment myocardial perfusion during cardiac ischaemia. Indeed, we observe a substantial increase in coronary flow rate when we expose isolated perfused hearts to 10 mM L‐lactate (Figure 6A,C). Local mechanisms of metabolic blood flow regulation secondary to increased cardiac oxidative demand^36^ are expected to add to the elevated coronary perfusion induced by direct L‐lactate‐mediated arterial relaxation. The L‐lactate‐induced venous responses are even more heterogeneous, with rat hindlimb veins consistently contracting in response to low physiological L‐lactate concentrations (1 mM, Figure 4A–C), front limb veins showing no significant response even to 20 mM L‐lactate (Figure 4O–Q), and mesenteric veins relaxing but only at markedly elevated L‐lactate levels (20 mM, Figure 4L–N). The heterogeneity in vascular responsiveness between organs will allow L‐lactate to redistribute blood during conditions of acute cardiovascular stress.

H^+^ concentrations usually increase during endogenous L‐lactate production, resulting in local or systemic metabolic acidosis. The metabolism of L‐lactate in other tissues and compensation by hyperventilation will minimize the decrease in arterial blood pH. It has been known for more than a century that alterations in extracellular pH (pH_o_) modulate arterial tone,^2^ whereas the functional effects of the conjugate bases accumulating in parallel with H^+^ are much less described.^37^ In this study, we adjusted all salt solutions used for ex vivo experiments to pH 7.40 to isolate the vascular and cardiac effects of L‐lactate and other carboxylates from the effects associated with changes in pH_o_.^38^ Depending on the magnitude and duration, intracellular acidification can cause either contraction or relaxation of blood vessels^39^ and can inhibit left ventricular contractile function.^40^, ^41^ H^+^‐linked uptake of lactate via monocarboxylate transporters^42^ will reduce pH_i_, but we observe only marginal (0.03 pH‐units magnitude) and transient intracellular acidification of the vascular smooth muscle cells in rat coronary septal arteries in response to 10 and 30 mM L‐lactate (Figure 3F). These observations—and similar reports of modest reductions in pH_i_ when rat mesenteric arteries are exposed to 50 mM Na‐L‐lactate^7^—suggest that H^+^‐linked uptake of L‐lactate in vascular smooth muscle cells is relatively limited or that L‐lactate entering the intracellular space is rapidly metabolized.

The positive inotropic effect of L‐lactate is revealed in vivo by an increase in left ventricular ejection fraction (Figure 7D) and in isolated perfused hearts by elevated left ventricular developed pressure at constant pre‐ and afterload (Figure 6B,C). At rest, the myocardium predominantly relies on fatty acids for oxidative phosphorylation^43^ but lactate replaces fatty acids in response to increased myocardial work.^44^ Determined by concentration gradients created by fermentative glycolysis and mitochondrial respiration, L‐lactate is shunted from cells with high glycolytic activity to cells with available oxidative capacity.^13^

In the current study, we did not explore the effects of the carboxylate metabolites at different pH levels. In most arterial beds, a dual vasorelaxant influence of H^+^ and the conjugate base is expected. However, responses to metabolites and H^+^ could interact, and there are also examples of arteries that contract when pH_o_ is lowered.^45^ Thus, the integrated responses are somewhat unpredictable and require future evaluation. Studies of venous actions of acid–base disturbances are missing, and investigations of lymphatic vessels are scarce, with the overall conclusion that acidosis inhibits lymphatic pumping activity.^46^, ^47^ In the heart, we expect the positive inotropic effect of L‐lactate will counteract the negative contractile influence of acidosis.^40^, ^41^

Increasing the osmolarity of experimental solutions influences vasomotor tone, particularly in some vascular beds (Figures 3H,K,N and 4B,M,P), but the effects of equiosmolar NaCl in most instances do not equal those of the carboxylate sodium salts (Figures 1, 2, 3, 4, 5). Also, whereas L‐lactate relaxes arteries and preferentially contracts veins, the effect of D‐lactate in both regards is much attenuated (Figures 3D,E and 4D,E). These observations contrast with a previous report^31^ proposing that changes in osmolarity mediate the effects of lactate on feeding behavior and thermogenesis. Also, the stereospecific nature of the response of isolated blood vessels to L‐lactate is different from the equal vasomotor actions of D‐ and L‐3‐hydroxybutyrate.^5^, ^48^

L‐lactate, DL‐3‐hydroxybutyrate, and butyrate fit on a continuum from preferential venocontraction to prominent venorelaxation (Figure 2C,D,F and Figure S4). Infusions to reach the cardiovascular active concentration range of DL‐3‐hydroxybutyrate—that begins at 1–3 mM with an EC_50_ of 12.4 mM^5^—also elevate blood and plasma levels of lactate substantially above NaCl‐treated rats (3.4 ± 0.6 mM vs 2.0 ± 0.2 mM; Figure 7A,B). In contrast, cardiovascular meaningful doses of butyrate show no effect on in vivo levels of lactate (Figure 7A,C). Lactate accumulation during ketosis occurs most likely due to reduced lactate oxidation^49^ and because conversion of 3‐hydroxybutyrate to acetyl coenzyme A limits consumption of pyruvate in the tricarboxylic acid cycle and thereby diverts it toward lactate production. Whereas butyrate infusion did not elevate lactate levels, it modestly increased the concentration of 3‐hydroxybutyrate, which can be explained by its role as a ketogenic substrate as well as by its ability to promote—via inhibition of histone deacetylase 3—release of fibroblast growth factor 21 that in turn stimulates hepatic ketogenesis.^50^ Accumulation of 3‐hydroxybutyrate is also favored by higher affinity of butyrate for entry into mitochondrial metabolism especially in the failing heart, which may displace 3‐hydroxybutyrate from oxidation.^51^ We propose that venorelaxation in response to butyrate infusion, mixed venorelaxation and ‐contraction in response to DL‐3‐hydroxybutyrate infusion with associated lactate accumulation, and preferential venocontraction to L‐lactate infusion explain the trend of gradually increasing left ventricular end‐diastolic volume (Figure 7D) in response to these carboxylates. In the current study, we tested acute influences—occurring within minutes of carboxylate administration—on vasomotor function, cardiac filling, and performance. In addition to fluid volume shifts in response to altered venous compliance, the different patterns of altered arterial and venous tone (Figures 1 and 2) are expected to change the balance between pre‐ and postcapillary resistance, which controls capillary hydrostatic pressure and fluid filtration to the interstitial space. Interestingly, the expected increase in fluid filtration in response to L‐lactate may also enhance macromolecular fluxes and possibly nutrient delivery through solvent drag,^52^ although the magnitude and impact of this effect is unclear.

We used similar infusion protocols for L‐lactate, DL‐3‐hydroxybutyrate, and butyrate. If we assume that the infused carboxylates distribute evenly in extracellular water (around 24% of body weight for rats),^53^ the plasma concentrations are expected to rise by 6.9 mM during the 10 min of infusion. Our measurements show that the concentrations increase more modestly by 1.6 mM lactate, 3.0 mM 3‐hydroxybutyrate, and 2.4 mM butyrate. These observations suggest that around 77% of infused L‐lactate, 57% of infused DL‐3‐hydroxybutyrate, and 65% of infused butyrate are taken up by cells, metabolized, or excreted within the 10‐min observation period.

From a clinical perspective, the ability of L‐lactate to enhance cardiac output and stroke volume without increasing heart rate may be leveraged to support acute patient care. Controlled infusions of L‐lactate could improve cardiac contractile function in conditions without excessive strain on the heart and aid in hemodynamic stabilization. In addition, the dual effects of arterial relaxation and selective venous contraction could help manage blood distribution in critically ill patients, including those with sepsis or trauma, by improving preload and diverting blood flow to the coronary circulation. In a rehabilitation setting, the targeted blood flow redistribution may increase oxygen delivery to active muscles to enhance exercise tolerance. These insights position L‐lactate as an important biological signal for cardiovascular adaptation and a potential therapeutic tool across cardiovascular care. Next steps include studies of in vivo hemodynamic responses in humans and identification of involved receptors and downstream effectors.

## 4 MATERIALS AND METHODS

We investigated cardiovascular responses to nine carboxylate metabolites: L‐lactate, DL‐3‐hydroxybutyrate, 3‐hydroxypropionate, pyruvate, acetate, propionate, butyrate, malonate, and succinate. Chemical structures were drawn using ChemSketch software (2024.2.0; ACD/Labs). We added the carboxylates as sodium salts, and to control for influences of added Na^+^ and changes in osmolarity, throughout the study, we compared the effects of sodium carboxylate salts to effects of equiosmolar NaCl. In a few select cases, we compared L‐ and D‐lactate; and in order to cover a larger concentration range that allows for the construction of full concentration‐response curves without effects on osmolarity, we substituted in these cases Na‐lactate for NaCl. We used the racemic mixture of Na‐DL‐3‐hydroxybutyrate because previous studies show that the two enantiomers have equivalent actions on isolated arteries.^5^

### 4.1 Rats

Male Sprague–Dawley rats from Janvier Labs or Taconic Biosciences were housed at Aarhus University under a 12‐h light/12‐h dark cycle with ad libitum access to water and standard chow. Rats were studied at ages between 10 and 16 weeks and acclimatized for at least 7 days before experiments.

### 4.2 Human biopsies

We obtained human blood vessel segments (left internal mammary arteries, internal thoracic veins, and great saphenous veins) from patients undergoing CABG or JOPCAB procedures at the Department of Cardiothoracic and Vascular Surgery, Aarhus University Hospital, Denmark. The Mid‐Jutland Regional Committee on Health Research Ethics approved the procedure for sampling human blood vessels (1‐10‐72‐128‐20), and all included patients gave written informed consent. We report the clinical characteristics of the included patients in Table 1.

### 4.3 Solutions

The PSS used for wire myograph experiments consisted of the following (in mM),^54^ 119 NaCl, 22 NaHCO_3_, 10 HEPES, 1.2 MgSO_4_, 2.82 KCl, 5.5 glucose, 1.18 KH_2_PO_4_, 0.03 ethylenediaminetetraacetic acid (EDTA), and 1.6 CaCl_2_. The Krebs–Henseleit (KH) solution used for experiments on isolated perfused hearts consisted of (in mM): 118.5 NaCl, 22 NaHCO_3_, 1.2 MgSO_4_, 4.7 KCl, 11 glucose, 1.2 KH_2_PO_4_, and 2.4 CaCl_2_. The supraphysiological glucose concentration of the KH buffer was chosen to ensure adequate metabolic substrate delivery in the absence of insulin and fatty acids.^55^ Solutions with elevated K^+^ concentration were prepared by equimolar substitution of KCl for NaCl. For ex vivo experiments, NaCl or sodium salts of the investigated carboxylates were dissolved in PSS or KH solution that was heated to 37°C and aerated with gas mixtures of 5% CO_2_/balance O_2_ (KH buffer used for isolated perfused hearts) or 5% CO_2_/balance air (PSS used for blood vessel myography) before pH was adjusted to 7.40 using NaOH or HCl.

### 4.4 Isometric wire myography

Rats under deep CO_2_ or sevoflurane anesthesia were killed by cervical dislocation and exsanguination. Excised rat tissues were transferred immediately to ice‐cold PSS for isolation of blood vessels under a stereomicroscope.^4^ From rats, we evaluated coronary septal, caudal femoral, middle cerebral, renal interlobar, and mesenteric arteries as well as caudal femoral, saphenous, lateral marginal, profound brachial, and mesenteric veins mounted on 40‐μm stainless steel wires. Segments of human left internal mammary arteries, internal thoracic veins, and great saphenous veins were transferred in ice‐cold PSS from the surgical department to the laboratory for experimental exploration. The human blood vessels were cleaned of excess perivascular tissue under a stereomicroscope and mounted on 200‐μm tungsten pins (DMT).

After heating to 37°C in PSS‐filled myograph chambers (DMT 610M and 620M), and during continuous bubbling with 5% CO_2_/balance air, arteries were normalized to 90% of the internal diameter corresponding to a transmural pressure of 100 mmHg.^56^ Veins were normalized to an internal diameter corresponding to a transmural pressure of 20 mmHg.^57^

Rat blood vessels were exposed to a standard warm‐up protocol consisting of five 1‐min long contractions elicited by 60 mM extracellular K^+^. Subsequently, the initial maximal contraction was determined by increasing the extracellular K^+^ concentration to 120 mM in the presence of 0.1 μM thromboxane analog U46619. Unless otherwise specified, relaxation and contraction responses were tested on rat blood vessels pre‐contracted with U46619 to a stable tension equivalent to around 60% of the initial maximal contraction, and vascular responses are reported relative to this pre‐contraction level. Blood vessels requiring more than 1 μM U46619 to reach the desired pre‐contraction level were excluded. We used U46619 for pre‐contraction because it shows a very uniform and stable response across blood vessels from different vascular beds, thus providing a reliable basis for comparison of metabolite responses between arteries and veins from different sources.

Human blood vessels were exposed to a warm‐up procedure consisting of two 10‐min long contractions elicited by 10 nM U46619. Responses to Na‐L‐Lactate, Na‐DL‐3‐hydroxybutyrate, and NaCl were tested in human arteries and veins pre‐contracted to at least 20% of the maximal contraction obtained during the warm‐up protocol, and vascular responses are reported relative to this pre‐contraction level. Depending on the size of the tissue biopsy, we mounted from every patient up to four segments of each blood vessel type.

Responses to the carboxylates were tested over 10 or 15 min. Arterial responses were averaged over the last 5 min and venous responses over the last 9 min of sodium carboxylate or NaCl exposure. If multiple blood vessels were tested from the same individual, or blood vessels were sequentially exposed to multiple sodium carboxylates or different concentrations of carboxylate sodium salts and NaCl, we alternated the order of exposure between experiments to control for influences of time.

### 4.5 Intracellular pH measurements

Using previously described protocols,^58^ we loaded coronary septal arteries mounted in wire myographs with 2 μM of the acetoxymethyl (AM) ester form of the pH‐sensitive fluorophore 2′,7′‐bis‐(2‐carboxyethyl)‐5‐(and‐6)‐carboxyfluorescein (BCECF; Thermo Fisher Scientific) solubilized in 0.01% dimethyl sulfoxide. Arteries maintained at 37°C were excited alternately at 440 and 495 nm, and emission light was collected at 530 nm using a Photon Technology International DeltaScan system with photomultiplier‐based detection. Background fluorescence measured before fluorophore loading was subtracted from all recorded emission values. Application of BCECF‐AM to the myograph bath solution was previously found to load vascular smooth muscle cells in the wall of resistance arteries with no detectable loading of endothelial cells.^59^ The BCECF fluorescence ratio (F_495_/F_440_) was calibrated to pH values using the high‐[K^+^] nigericin technique.^60^

### 4.6 Isolated perfused hearts

Rat hearts were isolated and retrogradely perfused as previously described.^5^ Briefly, following induction of anesthesia by a subcutaneous injection of Hypnorm‐Dormicum (fentanyl citrate, 0.158 mg/kg body weight; fluanisone, 0.5 mg/kg body weight; and midazolam, 0.5 mg/kg body weight), mechanical ventilation was initiated through a tracheostomy and a 500 IU bolus of heparin for anticoagulation was administered through the femoral vein. Through a thoracoabdominal incision, the ascending aorta was cannulated, and retrograde perfusion of the heart was initiated in situ using a 37°C, continuously aerated (5% CO_2_/balance O_2_) KH solution delivered at a constant pressure of 80 mmHg. After transfer to the Langendorff system (Harvard Apparatus) and following insertion of a water‐filled balloon in the left ventricle for continuous pressure recordings, preload was simulated by pressurizing the balloon to 5–8 mmHg. The hearts were then allowed to stabilize for 45 min before we tested the impact of adding 10 mM Na‐L‐lactate or 10 mM extra NaCl to the KH solution. Each isolated heart was exposed to one buffer change only.

We report cardiac parameters measured 10 min after buffer change and subtracted the response to equiosmolar NaCl. Hearts failing to meet the following inclusion criteria, adjusted for rat size, during the stabilization phase were excluded from the study^55^: coronary flow rate: 10–35 mL/min, arrhythmias: no sustained ventricular tachycardia or fibrillation, heart rate: 150–400 min^−1^, and left ventricular systolic pressure: >120 mmHg.

### 4.7 In vivo experiments

Solutions for in vivo administration were prepared by dissolving Na‐L‐lactate, Na‐DL‐3‐hydroxybutyrate, Na‐butyrate, or NaCl at 1 M in Milli‐Q water. These infusion solutions were adjusted to pH 7.0 at 37°C and administered intravenously using a Graseby 3500 syringe pump at a rate of 10 mL/h per kg body weight for 10 min.

The rats were immobilized in a supine position on a heating pad with integrated ECG electrodes, and body temperature was evaluated using a rectal probe and kept constant at 37°C throughout the experiment. After instrumentation, all rats received 1 mL isotonic NaCl via a venous catheter and were stabilized for at least 5 min before baseline parameters were acquired.

Echocardiography variables and simultaneous blood pressure were measured in sedated rats (3.25%–3.75% sevoflurane in air) intubated and ventilated mechanically (Ugo Basile 7025 ventilator) to an end‐tidal CO_2_ fraction of 5% measured with a Harvard Apparatus Type 340 capnograph. Heparin (50 IU) was administered by intramuscular injection to prevent coagulation, and sodium carboxylates and NaCl were administered via a catheter in the left femoral vein. Arterial blood pressures were measured with a solid‐state pressure catheter (Millar, SPR‐671) inserted in the right carotid artery, and cardiac dimensions were evaluated by transthoracic echocardiography (Fujifilm VisualSonics, Vevo 2100). Arterial blood was sampled at the end of the experiment from a catheter placed in the left femoral artery.

Echocardiography variables without simultaneous blood pressure were evaluated in rats sedated with 2.5% sevoflurane in air. Sodium carboxylates or NaCl were administered via a catheter in the tail vein, and at the end of the experiment, arterial blood was sampled from the left ventricle or the tail artery. Cardiac dimensions were evaluated by transthoracic echocardiography.

Blood pressure data were analyzed using LabChart 8 (AD Instruments), and echocardiography images were assessed using Vevo Lab software (Fujifilm VisualSonics). Left ventricular volumes were calculated using the bullet method.^61^, ^62^

### 4.8 Blood samples and carboxylate measurements

Blood was analyzed for L‐lactate concentration and pH using ABL800 or ABL90 FLEX blood gas analyzers (Radiometer). Alternatively, lactate, 3‐hydroxybutyrate, and butyrate were extracted from plasma with a mixture of methanol and acetonitrile, and their concentrations were measured by liquid chromatography tandem mass spectrometry (LC–MS/MS) using stable isotope‐labeled compounds as internal standards. Briefly, 25 μL plasma was added to 25 μL internal standard solution (500 μM lactate‐D_3_, ^13^C_4_‐3‐hydroxybutyrate, and ^13^C_4_‐butyrate in methanol), 25 μL solvent A (water:methanol 98:2 with 0.013% acetic acid), and 250 μL methanol:acetonitrile (1:1). After vortex mixing and centrifugation for 5 min at 10000 *g*, a 50‐μL aliquot of the supernatant was diluted with 150 μL solvent A, and 10 μL of this mixture was injected and analyzed for butyrate content by LC–MS/MS.^6^ Another 100‐μL aliquot of supernatant was used for quantification of lactate and 3‐hydroxybutyrate by LC–MS/MS essentially as previously described.^63^

### 4.9 Statistics

Normally distributed continuous data are presented as mean ± SEM, unless otherwise stated. Error bars are omitted if the SEM is smaller than the width of the symbol. We considered *p*‐values below 0.05 statistically significant. The reported *n*‐values represent biological replicates; and if multiple blood vessels from one individual were exposed to the same intervention, the average of these responses was calculated and counted as *n* = 1. Sample sizes were chosen based on previous experience using similar methodologies.^5^, ^6^ Within each batch of in vivo experiments, the recordings were analyzed by investigators blinded to the interventions. Single variables were compared between two groups using two‐tailed Student's *t*‐test and more than two groups using one‐way ANOVA followed by Dunnett or Šídák's posttest. We log‐transformed right‐skewed data or used Welch's correction to compare groups with unequal variances. If distributions remained non‐Gaussian after transformation, we performed the nonparametric Wilcoxon matched‐pairs signed rank test. The influence of two independent variables on a third variable was evaluated using two‐way ANOVA followed by Šídák's posttest. If variation was unequal between groups, we used Brown–Forsythe and Welch ANOVA tests. The statistical analyses were carried out using GraphPad Prism 10.1.1 software.

### 4.10 Ethics

All animal experimental procedures were approved by the Danish Animal Experiments Inspectorate (2018‐15‐0201‐01475 and 2021‐15‐0201‐00986). Human blood vessels were sampled from patients with ischaemic heart disease, who gave written informed consent, based on procedures approved by the Mid‐Jutland Regional Committee on Health Research Ethics (1–10–72‐128‐20).

## 5 CONCLUSION

Carboxylate metabolites generally cause arterial and venous relaxation, but the potency of different metabolites varies between vascular beds. L‐lactate stands out with a unique hemodynamic profile involving direct arterial relaxation accompanied by reduced systemic vascular resistance, venocontraction with associated elevated preload, and stimulation of cardiac contractility. Together, these effects can explain how exogenous administration of L‐lactate—through mechanisms independent of osmolarity and pH—enhances cardiac output without substantially influencing blood pressure or heart rate. We propose that L‐lactate optimizes hemodynamic function during metabolic disturbances and could benefit patients with a compromised cardiovascular system.

## AUTHOR CONTRIBUTIONS

**Casper Homilius:** Conceptualization; formal analysis; investigation; writing – review and editing; visualization; methodology; writing – original draft. **Jacob M. Seefeldt:** Conceptualization; formal analysis; investigation; visualization; writing – review and editing; methodology; writing – original draft. **Jakob Hansen:** Investigation; writing – review and editing. **Roni Nielsen:** Supervision; writing – review and editing. **Frank V. de Paoli:** Writing – review and editing; resources. **Ebbe Boedtkjer:** Conceptualization; funding acquisition; formal analysis; project administration; supervision; visualization; writing – original draft; writing – review and editing; methodology.

## FUNDING INFORMATION

This work was financially supported by the Novo Nordisk Foundation (grant no. NNF21OC0071822 to EB), Fonden til Lægevidenskabens Fremme (grant no. L‐2022‐00211 to EB), and the Independent Research Fund Denmark (grant no. 0134‐00294B to JMS).

## CONFLICT OF INTEREST STATEMENT

The authors declare that they have no conflict of interest related to this study. The coauthor Roni Nielsen has been an investigator on studies involving the pharmaceutical companies Imbria, Medtrace, and Janssen.

## Supporting information


Data S1:

## Data Availability

The data that support the findings of this study are available from the corresponding author upon reasonable request.
