# Supplementary material for: Lactate orchestrates metabolic hemodynamic adaptations through a unique combination of venocontraction, artery relaxation, and positive inotropy
Source: Acta Physiol (Oxf). 2025 Apr 1;241(5):e70037. doi: 10.1111/apha.70037 (PMC11960580; doi:10.1111/apha.70037)
Supplement: Supplementary file 1 — Data S1: [file APHA-241-e70037-s001.pdf]

Supplementary Material for

Lactate orchestrates metabolic hemodynamic adaptations through a unique combination of venocontraction, artery relaxation, and positive inotropy

Casper Homilius[a,*], Jacob M. Seefeldt[b,c,*], Jakob Hansen[d], Roni Nielsen[b,c],
Frank V. de Paoli[a,e], Ebbe Boedtkjer[a]

*These authors contributed equally to this work

[a]Department of Biomedicine, Aarhus University, Aarhus, Denmark
[b]Department of Clinical Medicine, Aarhus University, Aarhus, Denmark
[c]Department of Cardiology, Aarhus University Hospital, Aarhus, Denmark
[d]Department of Forensic Medicine, Aarhus University, Aarhus, Denmark
[e]Department of Cardiothoracic and Vascular Surgery, Aarhus University Hospital, Aarhus, Denmark

	Baseline characteristics, isolated blood vessels	
	Lumen diameter (µm)	Initial maximal tension (N/m)
Rat arteries		
Coronary septal	288±10	3.36±0.24
Caudal femoral	204±9	3.55±0.43
Middle cerebral	233±5	2.46±0.21
Mesenteric	289±17	7.76±0.39
Renal interlobar	381±25	3.71±0.53
Human arteries		
Left internal mammary	1807±63	19.39±2.27
Rat veins		
Caudal femoral	391±13	1.39±0.07
Profound brachial	676±24	2.23±0.17
Mesenteric	318±32	1.61±0.07
Lateral marginal	418±79	1.87±0.19
Saphenous	558±53	2.26±0.50
Human veins		
Internal thoracic	1610±138	5.86±0.94
Saphenous	3316±242	18.80±2.62

Supplementary Table S1. Diameters and initial maximal tension development elicited by 120 mM extracellular K^+ in combination with 0.1 µM U46619 are summarized for each of the evaluated blood vessel types. From rats, we evaluated 28 coronary septal artery segments, 15 caudal femoral artery segments, 15 middle cerebral artery segments, 10 mesenteric artery segments, 10 renal interlobar artery segments, 33 caudal femoral vein segments, 11 profound brachial vein segments, 8 mesenteric vein segments, 5 lateral marginal vein segments and 5 saphenous vein segments. From humans, we evaluated 13 left internal mammary artery segments, 16 internal thoracic vein segments, and 25 saphenous vein segments. The data represent mean±SEM and were compared using unpaired two-tailed Student's *t*-tests.

	Baseline characteristics, isolated perfused hearts		
	NaCl	Na-L-lactate	*P*-value
Left ventricular systolic pressure (mmHg)	163±4	166±4	0.64
Left ventricular developed pressure (mmHg)	157±4	160±4	0.55
Heart rate (min^{-1})	242±21	209±15	0.21
Coronary flow rate (mL/min)	17±2	18±2	0.70

Supplementary Table S2. Baseline values recorded at the end of the stabilization period before initiation of treatment from *ex vivo* experiments on isolated hearts (n=8-10). The data are mean±SEM and were compared using unpaired two-tailed Student's *t*-tests.

	Baseline characteristics, *in vivo* hemodynamics						
	NaCl	Na-L-lactate	*P*-value (*vs.* NaCl)	Na-DL-3-hydroxybutyrate	*P*-value (*vs.* NaCl)	Na-butyrate	*P*-value (*vs.* NaCl)
Body weight (g)	461±30	453±27	0.99	509±34	0.63	466±25	0.99
Cardiac output (mL/min)	144±8	135±9	0.76	160±5	0.52	160±5	0.53
Ejection fraction (%)	63±1	61±1	0.73	62±2	0.93	64±1	0.88
Heart rate (min^{-1})	337±10	318±7	0.34	338±12	0.99	317±16	0.51
Stroke volume (µL)	432±34	424±29	0.99	474±14	0.71	508±15	0.30
End-diastolic volume (µL)	679±46	686±38	0.99	768±20	0.39	793±29	0.24
End-systolic volume (µL)	251±16	261±12	0.90	294±17	0.18	284±17	0.40
Mean arterial pressure (mmHg)	93±3	98±6	0.46				
Systolic blood pressure (mmHg)	103±3	112±6	0.22				
Diastolic blood pressure (mmHg)	81±3	84±6	0.72				
Systemic vascular resistance (mmHg·min/mL)	0.83±0.06	0.97±0.06	0.11				

Supplementary Table S3. Baseline values before initiation of treatment from the *in vivo* echocardiography and blood pressure (n=6-15) measurements in Figure 7. The data represent mean±SEM and were compared using unpaired two-tailed Student's *t*-test (blood pressure and systemic vascular resistance) or one-way ANOVA followed by Dunnett's post-test (echocardiography and body weight).

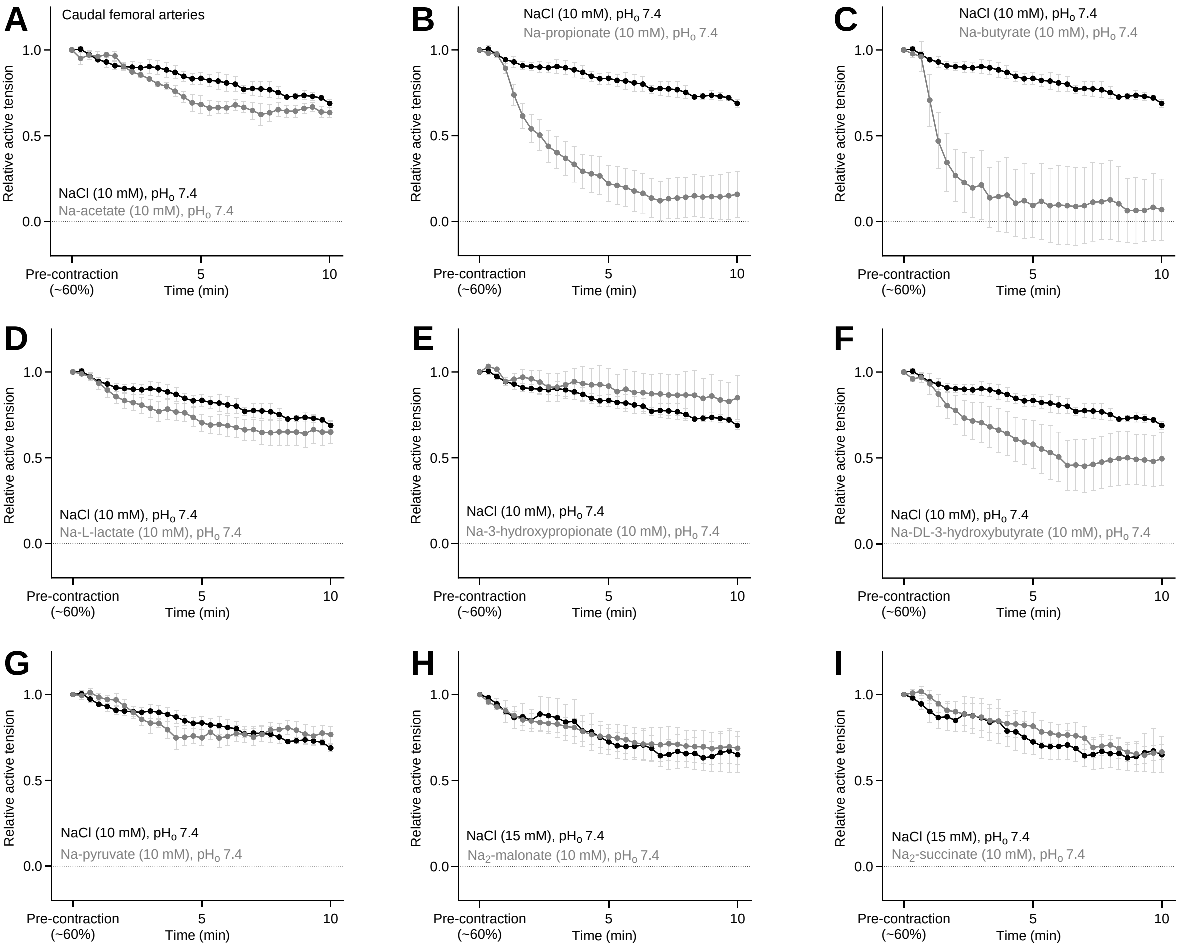

Supplementary Figure S1. Metabolic and microbial carboxylates relax rat caudal femoral arteries. The arterial responses are shown relative to a stable U46619-induced pre-contraction corresponding to around 60% of the initial maximal contraction. **A-I.** Average vasomotor responses of rat caudal femoral arteries mounted for evaluation of isometric tension development and stimulated with 10 mM of one of nine biologically prominent sodium carboxylates. For comparison, each panel repeats the response to equiosmolar NaCl (n=3-5). Summarized data are shown in Figure 1K. In all cases, pH of the buffer in the myograph bath was maintained by titrating the carboxylate-containing solutions to pH 7.40 before application.

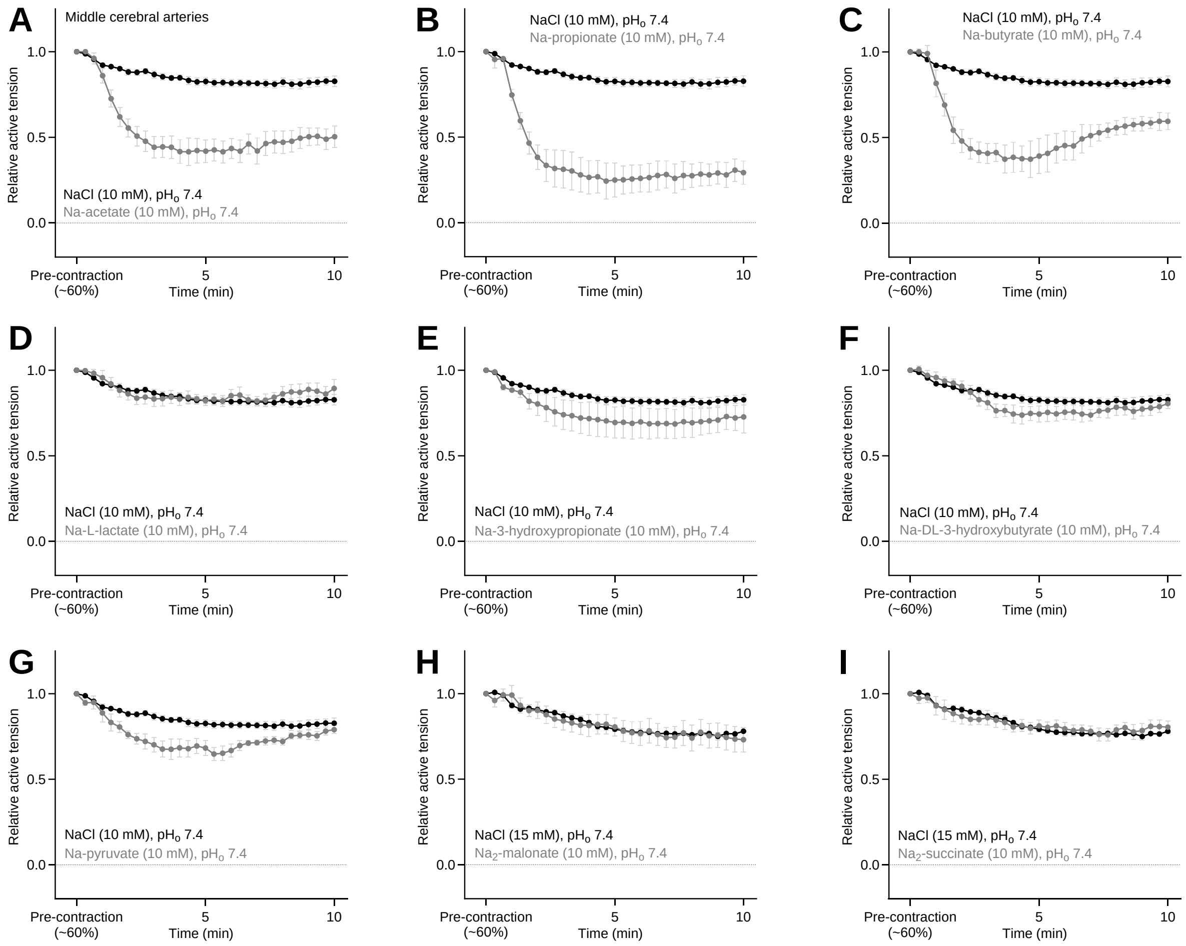

Supplementary Figure S2. Metabolic and microbial carboxylates relax rat middle cerebral arteries. The arterial responses are shown relative to a stable U46619-induced pre-contraction corresponding to around 60% of the initial maximal contraction. **A-I.** Average vasomotor responses of rat middle cerebral arteries mounted for evaluation of isometric tension development and stimulated with 10 mM of one of nine biologically prominent sodium carboxylates. For comparison, each panel repeats the response to equiosmolar NaCl (n=3-5). Summarized data are shown in Figure 1L. In all cases, pH of the buffer in the myograph bath was maintained by titrating the carboxylate-containing solutions to pH 7.40 before application.

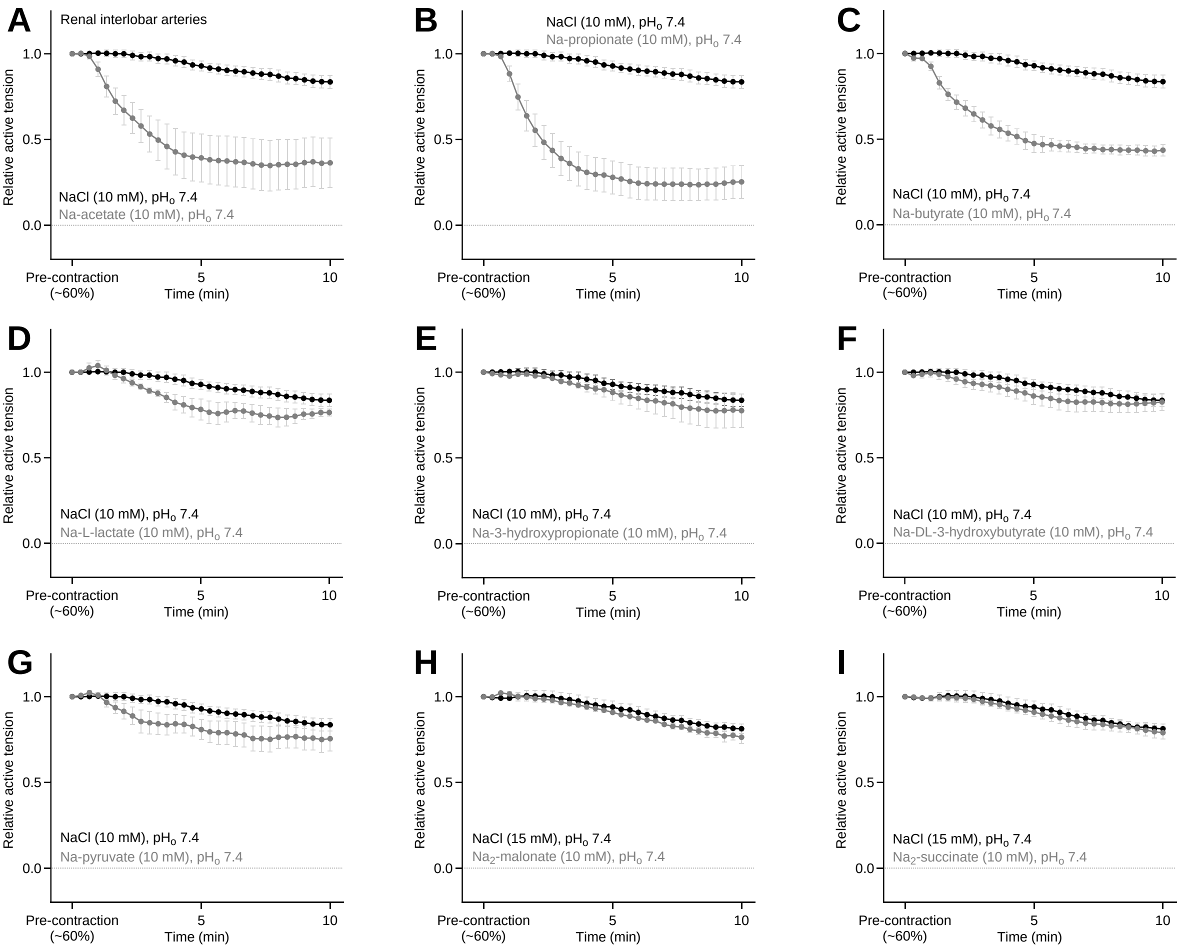

Supplementary Figure S3. Metabolic and microbial carboxylates relax rat renal interlobar arteries. The arterial responses are shown relative to a stable U46619-induced pre-contraction corresponding to around 60% of the initial maximal contraction. **A-I.** Average vasomotor responses of rat renal interlobar arteries mounted for evaluation of isometric tension development and stimulated with 10 mM of one of nine biologically prominent sodium carboxylates. For comparison, each panel repeats the response to equiosmolar NaCl (n=2-5). Summarized data are shown in Figure 1M. In all cases, pH of the buffer in the myograph bath was maintained by titrating the carboxylate-containing solutions to pH 7.40 before application.

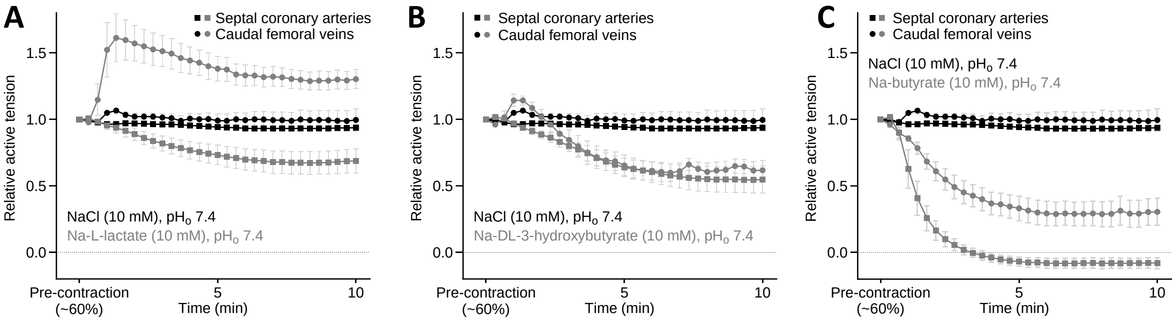

Supplementary Figure S4. Summary of the arterial and venous responses to L-lactate, DL-3-hydroxybutyrate, and butyrate. The arterial and venous responses are shown relative to a stable U46619-induced pre-contraction corresponding to around 60% of the initial maximal contraction. **A-C.** Average responses of coronary septal arteries and caudal femoral veins to 10 mM Na-L-lactate (A, n=5), Na-DL-3-hydroxybutyrate (B, n=5), and Na-butyrate (C, n=4). Each panel also shows the corresponding response to equiosmolar NaCl for comparison (n=4-5). The figure repeats data from Figures 1 and 2 to summarize the different responses to the three carboxylates. In all cases, pH of the buffer in the myograph bath was maintained by titrating the carboxylate-containing solutions to pH 7.40 before application.
